# Supplementary material for: Exhaled nitric oxide detection for diagnosis of COVID-19 in critically ill patients
Source: PLoS One. 2021 Oct 28;16(10):e0257644. doi: 10.1371/journal.pone.0257644 (PMC8553051; doi:10.1371/journal.pone.0257644)
Supplement: S1 File — (PDF) [file pone.0257644.s001.pdf]

Diagnosis	Patient	Day	Type of response	Vent Day	P/F ratio (closest to sample)	S/F ratio	FiO2 (at time of sample collection)	PEEP (at time of sample collection)	GCS	CV - lowest MAP	CV - highest vasopressor	Bilirubin	Platelets	Renal Replacement Therapy	Creat	BUN	Highest Temperature	WBC	Lymphocyte Count	Sens Ind NC	ns Ind NH3/	Ind: Omeg	FA Resp(P/	FA Resp(S/	SOFA Heme	SOFA Hep	OFA Cardi	SOFA Neur	SOFA Renal	Total SOFA	Past Medical History (Y / N)								
																															Heart Dz	Lung Dz	HTN	Obesity	DM	Cancer	Cirrhosis	ESRD	Survival
Control	1	1	NO	Y	273		30	6	7	72		3.0	61	N	1.63	37	103.1	4.06	1.38	0.509			2	0	2	2	0	3	1	10	1	0	1	0	0	0	0	0	0
Control	1	3	NO	Y	273		30	6	7	52		2.8	22	N	2.08	59	101.7	3.02	0.99	0.338			2	0	3	2	1	3	2	13									
Control	1	7	NO	Y	N/A	333	30	6	7	66		2.8	14	Y	1.40	41	100.2	5.86	1.21	0.089			0	2	4	2	1	3	4	16									
Control	2	1	NO	Y	N/A	250	30	6	15	69		0.4	243	N	0.50	22	98.7	12.66	1.03	2.314			0	2	0	0	1	0	0	3	1	1	1	0	0	1	0	0	1
Control	3	1	NO	Y	N/A	250	40	6	15	91		0.8	124	N	8.48	83	98.4	18.11	0.54	0.670			0	2	1	0	0	0	4	7	1	1	1	1	1	0	0	1	1
Control	4	1	NO	Y	N/A	320	30	6	10	75		0.3	346	N	0.80	20	98.5	16.89	N/A	0.239			0	2	0	0	0	2	0	4	1	0	1	0	0	0	0	0	0
Control	4	3	NH3/O2	Y	N/A	286	35	6	8	54	0.03	0.3	292	N	0.81	21	98.9	13.26	N/A		-0.247		0	2	0	0	3	3	0	8									
Control	4	7	Omega	Y	N/A	333	30	6	3	48	0.06	0.3	197	N	N/A	N/A	99.3	11.25	N/A			0.053	0	2	0	0	3	4	4	13									
Control	4	10	NH3/O2	Y	N/A	310	30	6	7	75		0.3	175	N	N/A	N/A	99.1	9.15	N/A		-0.265		0	2	0	0	0	3	4	9									
Control	5	1	NO	Y	74		80	8	7	33	0.2	1.7	292	N	2.66	87	99.9	55.72	0.37	0.280			4	0	0	1	4	3	2	14	1	1	0	1	0	0	0	0	1
Control	5	3	Omega	N										Y								0.033	4	0	4	0	1	4	4	17									
Control	6	1	NO	Y	N/A	160	55	10	9	46	0.15	0.6	209	N	3.42	90	103.6	13.83	0.96	0.476			0	3	0	0	4	3	3	13	1	1	0	1	0	1	0	1	1
Control	6	3	NO																	0.403			4	0	4	0	1	4	0	13									
Covid	7	1	Omega	Y	217		30	8	7	61	0.3	0.5	138	N	2.16	40	99.6	17.92	1.42			0.046	2	0	1	0	4	3	2	12	0	0	1	0	0	0	0	0	1
Covid	7	3	NH3/O2	Y	223		30	6	9	63		0.6	109	Y	1.53	48	99.8	20.03	0		-0.045		2	0	1	0	1	3	4	11									
Covid	7	7	NO	Y	201		35	10	9	80		0.9	60	Y	1.88	43	99.5	19.87	0.70	0.210			2	0	2	0	0	3	4	11									
Covid	7	10	NO	Y	258		30	6	5	62		0.9	108	Y	2.08	37	100.4	20.10	0.46	0.530			2	0	1	0	1	4	4	12									
Covid	8	1	Omega	Y	235		40	8	6	75		0.4	208	N	1.89	67	102	9.20	0.34			0.157	2	0	0	0	0	3	1	6	0	0	0	0	1	0	0	0	1
Covid	8	3	Omega	Y	85		60	10	8	51		0.5	256	N	1.61	61	101.4	17.28	0.40			0.044	4	0	0	0	1	3	1	9									
Covid	8	7	Omega	Y	117		60	14	3	70		0.8	183	N	1.93	68	101.3	23.33	0.42			0.044	3	0	0	0	0	4	2	9									
Covid	8	10	Omega	Y	159		45	12	3	63		0.8	165	N	2.09	80	100.4	20.35	1.14			0.117	3	0	0	0	1	4	2	10									
Control	9	1	NO	Y	354		30	6	8	58	0.02	0.7	294	N	1.17	22	98.2	35.40	3.39	0.631			1	0	0	0	3	3	0	7	1	0	1	0	0	0	0	0	0
Control	9	3	NO	Y	N/A		28	6	9	72		0.7	349	N	1.10	27	98.8	24.05	6.90	0.062			0	4	0	0	0	3	0	7									
Control	10	1	Omega	Y	N/A	240	40	6	10	59	0.13	37.1	73	Y	2.97	21	98.4	11.56	N/A			0.046	0	3	2	4	4	2	4	19	0	0	0	0	0	0	1	0	0
Control	10	3	NH3/O2	Y	142		40	6	10	55	0.12	38.5	42	Y	2.50	28	98.4	21.03	N/A		-0.212		3	0	3	4	4	2	4	20									
Control	10	7	NO	Y	190		60	8	6	56	0.4	37.9	61	Y	4.26	71	105.3	62.05	11.79	0.219			3	0	2	4	4	3	4	20									
Control	10	10	NO	Y	N/A	235	30	5	4	54	0.12	33.3	31	Y	1.65	39	100.1	21.49	N/A	0.414			0	3	3	4	4	4	4	22									
Covid	11	1	NO	Y	N/A	248	40	6	9	67		0.4	178	Y	1.87	36	99.7	13.10	1.31	0.242			0	2	0	0	1	3	4	10	0	1	1	0	0	1	0	0	1
Control	12	1	NO	Y	151		50	12	7	74		1.1	298	N	0.86	7	100.2	7.26	N/A	0.647			3	0	0	0	0	3	0	6	0	0	0	1	0	0	0	0	1
Control	12	3	NO	Y	N/A	184	50	12	15	73		1.1	229	N	0.56	8	99.3	5.65	N/A	0.249			0	3	0	0	0	0	0	3									
Control	12	7	NO	Y	168		45	15	15	92		1.1	277	N	0.58	13	99.9	6.53	N/A	0.988			3	0	0	0	0	0	0	3									
Control	13	1	NO	Y	N/A	245	40	6	6	77		21.0	79	N	1.41	13	100	15.19	N/A	0.324			0	2	2	4	0	3	1	12	0	0	1	0	0	0	1	0	1
Control	14	1	NO	Y	113		50	8	12	74	0.08		136	N	0.42	10	98.5	8.70	N/A	0.044			3	0	1	0	3	2	0	9	0	1	1	0	0	0	0	0	1
Control	15	1	NO	Y	186		65	10	11	63		0.0	290	N	0.45	N/A	98.6	11.28	0.23	0.687			3	0	0	0	1	2	0	6	0	0	1	0	0	1	0	0	0
Control	15	3	NO	Y	189		40	6	11	66		0.3	245	N	0.41	33	98.4	8.76	0.15	0.351			3	0	0	0	1	2	0	6									
Covid	16	1	Omega	Y	195		50	14	10	58	0.04	1.4	254	N	1.05	39	39.4	7.9	0.62			0.224	3	0	0	1	3	2	0	9	0	0	1	1	1	0	0	0	1
Covid	16	3	NO	Y	250		100	10	6	62	0.02	0.8	231	N	1.07	70	37.9	7.28	0.39	0.2415			2	0	0	0	3	3	0	8									
Covid	16	7	NO	Y	240		40	8	11	49		0.8	216	N	0.72	42	37.8	9.62	0.42	0.9636			2	0	0	0	1	2	0	5									
Control	17	1	NO	Yes	N/A	184	50	10	10	74		1.8	8	Y/N	0.5	26	37.7	3.23	0	0.259			0	3	4	1	0	2	0	10	0	0	1	0	0	1	0	0	0
Control	17	3	Omega	Yes	N/A	188	40	8	10	74		1.6	5	Y/N	0.47	35	37.3	5.25	0			0.104	0	3	4	1	0	2	0	10									
Covid	18	1	Omega	Yes	N/A	182	45	8	8	66		1	137	No	0.95	30	37.1	7.54	0.28			0.112	0	3	1	0	1	3	0	8	1	0	1	0	1	0	0	1	1
Covid	18	3	Omega	Yes	N/A	173	40	8	8	75		0.6	152	No	0.91	45	38.3	5.9	0.11			0.039	0	3	0	0	0	3	0	6									
Covid	18	7	NO	Yes	N/A	196	40	6	3	64		0.9	162	No	1.06	82	38.5	10.56	0.42	0.210			0	3	0	0	1	4	0	8									
Covid	18	10	NH3/O2	Yes	N/A	240	40	6	8	66		0.9	131	No	0.78	72	37.8	13.76	0.3		-0.423		0	3	1	0	1	3	0	8									
Control	19	1	Omega	Yes	499.64		40	6	11	64		0.4	48	Yes	4.52	24	36.8	6.64	N/A			0.098	0	0	3	0	1	2	3	9	1	1	1	1	1	0	0	0	0
Control	19	3	NO	Yes	N/A	330	30	6	11	75			73	Yes	2.22	19	37.5	N/A	N/A	0.643			0	2	2	0	0	2	2	8									
Control	19	7	NO	Yes	127		30	6	10	63			145	Yes	2.03	21	36.9	7.91	N/A	0.066			3	0	1	0	1	2	2	9									
Control	19	10	sensor dead	Yes	N/A	250	40	6	13	57		0.7	211	Yes	2.07	31	36.9	7.15	N/A				0	2	0	0	1	1	2	6									
Control	20	1	NO	Yes	300.2		50	12	3	49	0.6	0.7	175	Yes	0.56	18	38.2	15.62	1.9	0.457			1	0	0	0	4	4	0	9	1	1	1	0	0	1	0	0	0
Control	20	7	NO	Yes	106.4		70	6	3	44	1.2		109	Yes	0.55	34	38.6	9.3	N/A	0.181			3	0	1	0	4	4	0	12									
Control	20	10	sensor dead	Yes	N/A	169	55	12	9	66		0.5	99	Yes	1.38	64	37.3	11.48	N/A				0	3	2	0	1	3	1	10									
Control	21	1	Omega	Yes	N/A	235	35	6	9	77		0.1	208	N/A	0.98	20	37.7	7.56	N/A			0.061	0	3	0	0	0	3	0	6	1	1	1	0	0	0	0	0	1
Control	22	1	NH3/O2	Yes	N/A	317	30	6	7	71		34	55	No	5.03	91	37.7	9.64	N/A		-0.352		0	2	2	4	0	3	4	15	0	0	1	0	0	0	1	0	0
Control	22	3	NO	Yes	N/A	313	30	6	7	52	0.18	41.7	72	Yes	2.43	42	37.5	18.55	N/A	0.186			0	2	2	4	4	3	2	17									
Covid	23	1	NO	Yes	N/A	90	40	10	11	113		0.2	192	Yes	2.46	43	36.7	25.26	N/A	0.271			0	4	0	0	0	2	2	8	0	0	1	1	1	0	0	1	1
Control	24	1	NO	Yes	169.57		45	10	3	66		1.9	28	No	2.68	50	37.1	<.30	N/A	0.852			3	0	3	2	1	4	2	15	0	0	1	1	0	1	0	0	1
Control	24	3	NO	Yes	217.75		40	10	10	55		1.5	23	Yes	4.94	102	37.2	2.22	0.1	0.237			2	0	3	1	1	2	4	13									
Covid	25	1	sensor dead	Yes	132.33		60	10	6	66		1.2	133	No	0.49	13	36.9	15.33	0.67				3	0	1	0	1	3	0	8	1	1	1	0	0	0	0	0	0
Covid	25	3	sensor dead	Yes	233.6		50	10	8	72		0.8	203	No	0.42	18	38.2	10.63	0.76				2	0	0	0	0	3	0	5									
Covid	25	7	sensor dead	Yes	193		50	10	3	68		0.6	209	No	0.4	20	37	6.99	0.94				3	0	0	0	1	4	0	8									
Covid	25	10	NO	Yes	138.6		70	12	3	61	0.04	0.6	149	No	0.53	25	37.8	15.17	0.81	2.306			3	0	1	0	3	4	0	11									
Covid	26	1	sensor dead	Yes	152.6		50	12	5	44	0.17	0.5	326	No	0.9	18	38.3	9.1	0.65				3	0	0	0	4	4	0	11	1	0	1	1	1	0	0	0	0
Covid	26	3	sensor dead	Yes	142.54		55	14	7	60	0.15	0.6	257	No	1.11	40	38.5	6.15	0.87				3	0	0	0	4	3	0	10									
Covid	26	7	NO	Yes	127.4		50	12	4	60	0.13	0.4	235	No	1.34	48	38.6	11.32	N/A				3	0	0	0	4	4	1	12									
Covid	26	10	Omega	Yes	83		80	14	7	57	0.12	0.4	236	No	1.44	44	38.2	10.85	N/A	0.752			4	0	0	0	4	3	1	12									
Covid	27	1	Omega	Y	102.9		65	16	3	81		0.5	210	N	0.64	17	37.3	21.18	0.64			0.166	3	0	0	0	0	4	0	7	0	0	1	1	1	0	0	0	1
Covid	27	3	nsor diff temp	Y	178.4		50	14	3	72		0.4	193	N	0.8	37	37.7	14.58	N/A			0.221	3	0	0	0	0	4	0	7									
Covid	27	7		Y	99.7		70	12	3	75		0.6	195	N	0.56	22	37.1	10.26	1.28				4	0	0	0	0	4	0	8									
Covid	27	10		Y	N/A		40	10	6	83		0.6	223	N	0.59	24	37.6	15.99	1.43				0	4	0	0	0	3	0	7									
Covid	28	1	low volume	Yes	234		40	12	9	51	0.05	0.5	329	Yes	3.76	53	97.6	37	N/A				2	0	0	0	3	3	3	11	1	0	1	0	1	0	0	1	1
	29	1	nsor diff temp																				4	0	4	0	1	4	0	13									
Covid	30	1	nsor diff temp	Yes	N/A	202	45	10	9	78		0.3	164	Y/N	2.73	82	98.4	8.29	0.09				0	3	0	0	0	3	2	8	1	0	1	1	1	0	0	1	1
Covid	30	3	nsor diff temp	Yes	N/A	223	40	6	8	87		0.2	161	Y/N	1.91	64	99.9	5.06	0.17				0	3	0	0	0	3	2	8									
Covid	30	7	nsor diff temp	Yes	113.85		100	12	8	62		0.3	141	Y/N	1.63	56	98.6	4.2	0.1				3	0	1	0	1	3	1	9									
Covid	31	1	nsor diff temp	Yes	115		60	18	3	63		0.7	382	No	1.19	37	100.6	10.46	N/A				3	0	0	0	1	4	0	8	0	0	1	0	1	0	0	0	0
Covid	31	3	nsor diff temp	Yes	92		70	14	6	64		0.4	315	No	0.81	32	99.7	9.61	N/A				4	0	0	0	1	3	0	8									
Covid	31	7	nsor diff temp	Yes	181		40	10	3	57		0.4	256	No	0.8	30	102.4	9.74	N/A				3	0	0	0	1	4	0	8									
Covid	31	10	nsor diff temp	Yes	92		70	12	9	50		0.5	216	No	0.62	25	101.1	7.07	N/A				4	0	0	0	1	3	0	8									
Covid	32	1	nsor diff temp	Yes	N/A	266	35	8	N/A	78		0.4	166	Yes	5.43	83	98.6	10.1	N/A				0	2	0	0	0	0	4	6	1	1	1	1	0	0	0	1	1
Covid	32	3	nsor diff temp	Yes	N/A	204	45	8	8	72			N/A	Yes	N/A	N/A	101.1	N/A	N/A				0	3	0	0	0	3	4	10									
Covid	32	7	nsor diff temp	Yes	N/A	228	40	6	11	80			213	Yes	3.14	95	100.8	11.75	N/A				0	3	0	0	0	2	2	7									
Covid	33	1	nsor diff temp	Yes	192.6		45	14	3	58	0.09	0.4	291	Y/N	0.64	27	100	23.62	0.85				3	0	0	0	3	4	0	10	0	0	1	0	0	0	0	0	1
Covid	33	3	nsor diff temp	Yes	156		50	10	4	63	0.02	0.4	231	Y/N	0.46	33	99.5	15.99	0.53				3	0	0	0	3	4	0	10									
Covid	33	7	nsor diff temp	Yes	143		60	16	3	67		0.4	155	Y/N	0.36	24	100.2	14.63	0.81				3	0	0	0	1	4	0	8									
Covid	33	10	Omega	Yes	185		60	12	3	68		0.4	149	Y/N	0.31	20	100	13.46	1.36				3	0	1	0	1	4	0	9									
Control	34	1	NO	Y	213		35	6	10	109	0.15	0.3	172	Y	1.51	31	99.5	20.27	N/A			0.166	2	0	0	0	4	2	4	12	1	1	1	0	1	0	0	1	0
Control	34	3	Omega	Y	N/A	323	30	6	9	99	0.09	0.3	130	N	2.96	52	99.5	13.86	N/A	2.575			0	2	1	0	3	3	2	11									
Control	34	7	Omega	Y	297		40	8	10	85	0.18	0.3	76	N	4.24	63	98.9	16.40	N/A			0.231	2	0	2	0	4	2	3	13									
Control	35	1	Omega	Y	N/A	97	40	8	5	75		4.3	107	N	1.69	N/A	97.7	N/A	N/A			0.175	0	4	1	2	0	4	1	12	0	1	0	1	0	0	1	0	1
Covid	36	1	Omega	Y	267		50	14	3	89	0.2	0.9	219	N	1.80	56	98.7	18.27	N/A			0.290	2	0	0	0	4	4	1	11	1	0	1	1	0	0	0	0	1
Covid	36	3	Omega	Y	245		100	16	3	87	0.06	0.6	251	N	2.61	94	99	24.77	N/A			0.553	2	0	0	0	3	4	2	11									
Covid	36	7	Omega	Y	161		50	12	7	83	0.01	0.6	190	N	2.37	92	98.8	29.93	N/A			0.278	3	0	0	0	3	3	2	11									
Covid	36	10	Omega	Y	218		35	8	9	96		0.6	89	N	1.68	51	98.9	16.47	N/A			0.109	2	0	2	0	0	3	1	8									
Covid	37	1	Omega	Y	N/A	182	50	10	3	109		0.5	29	Y	3.98	43	101.5	22.47	0.79			0.100	0	3	3	0	0	4	4	14	0	1	1	0	0	1	1	0	1
Covid	37	3	Omega	Y	98		40	8	4	101		0.7	55	Y	1.26	28	99.1	20.81	2.13			0.093	4	0	2	0	0	4	4	14									
Covid	38	1	Omega	Y	147		45	6	11	92		0.5	234	N	1.06	40	99.3	9.67	0.36			0.104	3	0	0	0	0	2	0	5	0	1	1	0	0	0	0	0	0
Covid	38	3	NO	Y	136		60	12	11	108		0.6	198	N	0.99	36	101.1	8.11	0.32	1.535			3	0	0	0	0	2	0	5									
Covid	38	7	Omega	Y	130		75	12	3	94		0.3	256	N	1.04	49	98.8	8.53	0.33			0.187	3	0	0	0	0	4	0	7									
Covid	39	1	Omega	Y	N/A	211	40	12	8	83	0.08	0.5	197	N	0.87	24	98.7	11.54	1.09			0.515	0	3	0	0	3	3	0	9	1	0	1	1	0	1	0	0	1
Covid	39	3	Omega	Y	N/A	307	30	6	11	121		0.3	205	N	0.64	34	98.5	12.95	1.57			0.668	0	2	0	0	0	2	0	4									
Covid	40	1	Omega	Y	N/A	190	40	8	11	74	0.08	0.5	298	N	1.60	43	98.2	12.24	1.03			0.104	0	3	0	0	3	2	1	9	0	0	1	1	0	0	0	0	1
Covid	40	3	Omega	Y	N/A	223	40	6	15	123		0.4	288	N	1.38	60	99.7	11.33	N/A			0.103	0	3	0	0	0	0	1	4									
Covid	41	1	Omega	Y	390		85	20	3	83	0.05	0.5	88	N	1.54	34	101.5	3.98	0.82			0.408	1	0	2	0	3	4	1	11	0	0	1	1	1	0	0	0	1
Covid	41	3	Omega	Y	140		100	12	3	89	0.06	0.3	132	N	1.44	50	100	5.57	0.34			0.155	3	0	1	0	3	4	1	12									

Covid	41	7	Omega	Y	204		40	10	10	82		0.6	224	N	0.85	37	100.2	8.93	1.04		0.089
Covid	41	10	Omega	Y	N/A	235	35	6	13	116		0.6	172	N	0.87	36	100	8.38	N/A		0.119
Covid	42	1	Omega	Y	129		70	14	3	119		0.3	253	N	1.81	58	99.1	10.90	0.20		0.608
Covid	42	3	Omega	Y	143		45	14	8	138		0.4	283	N	1.56	69	100.8	17.30	0.16		0.096
Covid	42	7	Omega	Y	143		40	6	11	106		0.4	129	N	1.85	76	100.9	21.59	0.82		0.195
Covid	43	1	Omega	Y	323		45	8	8	91		1.4	171	Y	4.08	42	100.2	11.35	0.35		0.549
Covid	43	3	Omega	Y	N/A	180	45	6	7	112		1.4	163	N	3.45	63	99.4	10.37	N/A		0.320
Covid	43	7	Omega	Y	N/A	218	45	6	6	104		1.5	163	Y	3.45	77	100	18.78	N/A		0.477
Covid	43	10	Omega	Y	N/A	303	30	6	9	91		1.7	155	Y	4.50	90	99	28.39	N/A		0.341
Covid	45	1	Omega	Y	116		100	14	3	84		0.4	135	N	1.34	35	98.9	11.32	0.78		0.544
Covid	45	3	Omega	Y	270		45	14	3	91		0.3	133	N	1.18	41	100	9.22	0.56		0.140
Covid	45	7	NO	Y	N/A	92	55	8	5	80		0.5	148	N	1.03	34	102	13.65	0.8		0.410
Control	46	1	NO	Y	67		90	14	7	87	0.16	0.4	141	N	4.51	85	98.3	12.06	0.21	2.105	
Control	46	7	NO	Y	119		45	8	8	95	0.03	0.4	78	Y	4.20	130	98.7	12.72	N/A	1.602	
Control	47	1	NO	Y	N/A	86	80	10	10	73	0.16	0.4	201	N	0.83	15	101.7	8.23	0.56	2.283	
Control	48	1	NO	Y	N/A	237.5	40	6	10	66		0.5	140	N	0.83	15	98.2	6.43	0.60	1.242	
Control	48	7	NO	Y	N/A	87	50	10	10	84		0.5	119	N	0.51	17	99.1	7.02	0.50	1.353	

Covid	41	7	2	0	0	0	0	2	0	4									
Covid	41	10	0	3	0	0	0	1	0	4									
Covid	42	1	3	0	0	0	0	4	1	8	0	1	0	1	0	0	0	0	1
Covid	42	3	3	0	0	0	0	3	1	7									
Covid	42	7	3	0	1	0	0	2	1	7									
Covid	43	1	1	0	0	1	0	3	4	9	1	0	1	1	1	0	0	1	0
Covid	43	3	0	3	0	1	0	3	3	10									
Covid	43	7	0	3	0	1	0	3	4	11									
Covid	43	10	0	2	0	1	0	3	4	10									
Covid	45	1	3	0	1	0	0	4	1	9	0	1	1	1	1	0	0	0	0
Covid	45	3	2	0	1	0	0	4	0	7									
Covid	45	7	0	4	1	0	0	4	0	9									
Control	46	1	4	0	1	0	4	3	3	15	1	1	1	1	0	0	0	0	1
Control	46	7	3	0	2	0	3	3	4	15									
Control	47	1	0	4	0	0	4	2	0	10	0	1	0	0	1	1	0	0	0
Control	48	1	0	3	1	0	1	2	0	7	1	1	1	0	1	1	0	0	1
Control	48	7	0	4	1	0	0	2	0	7									
