# Supplementary material for: Exhaled nitric oxide detection for diagnosis of COVID-19 in critically ill patients
Source: PLoS One. 2021 Oct 28;16(10):e0257644. doi: 10.1371/journal.pone.0257644 (PMC8553051; doi:10.1371/journal.pone.0257644)
Supplement: Attachment — Submitted filename: PONE-D-21-20035 Response to Reviewers.docx [file pone.0257644.s002.docx]

We thank the editors and reviewers for their thoughtful comments. We feel that their feedback has improved the quality of our work substantially. Please see below for specific responses.

**Response to PLOS :**

**When submitting your revision, we need you to address these additional requirements.**

**1. Please ensure that your manuscript meets PLOS ONE's style requirements, including those for file naming.**

We believe the resubmission meets style requirements.

**2. Thank you for including your ethics statement: "Institutional Research Board (IRB) and Institutional Biosafety Committee (IBC) approved study protocol according to local COVID-19 research protocols. Patients admitted to the intensive care unit (ICU) requiring mechanical ventilation participated in this study. Inclusion criteria included acute respiratory failure requiring endotracheal intubation and mechanical ventilation. Exclusion criteria included mechanical ventilation for > 72 hours, inability to obtain informed consent, or prisoner status. "**

**a) Please amend your current ethics statement to include the full name of the ethics committee/institutional review board(s) that approved your specific study.**

This has been updated.

**b) Please provide additional details regarding participant consent. In the ethics statement in the Methods and online submission information, please ensure that you have specified what type you obtained (for instance, written or verbal, and if verbal, how it was documented and witnessed). If your study included minors, state whether you obtained consent from parents or guardians. If the need for consent was waived by the ethics committee, please include this information.**

This has been updated.

**Once you have amended this/these statement(s) in the Methods section of the manuscript, please add the same text to the “Ethics Statement” field of the submission form (via “Edit Submission”).**

**3. Please modify the title to ensure that it is meeting PLOS’ guidelines (**[**https://journals.plos.org/plosone/s/submission-guidelines#loc-title**](https://urldefense.com/v3/__https:/journals.plos.org/plosone/s/submission-guidelines*loc-title__;Iw!!Cjnu1T6GFXg!5kUyB3tTfo_2QNeZFuMexc32vrxRJewf2OHX8H3xMvDnCc7i5AEkaKgEgkkLzbfKnPlNQQ$)**). In particular, the title should be "specific, descriptive, concise, and comprehensible to readers outside the field" and in this case we feel it is not informative and specific about your study's scope and methodology.**

We agree and have updated the title to be more descriptive.

**4. We note that you have a patent relating to material pertinent to this article. Please provide an amended statement of Competing Interests to declare this patent (with details including name and number), along with any other relevant declarations relating to employment, consultancy, patents, products in development or modified products etc. Please confirm that this does not alter your adherence to all PLOS ONE policies on sharing data and materials, as detailed online in our guide for authors** [**http://journals.plos.org/plosone/s/competing-interests**](https://urldefense.com/v3/__http:/journals.plos.org/plosone/s/competing-interests__;!!Cjnu1T6GFXg!5kUyB3tTfo_2QNeZFuMexc32vrxRJewf2OHX8H3xMvDnCc7i5AEkaKgEgkkLzbfOt5yGtQ$) **by including the following statement: "This does not alter our adherence to  PLOS ONE policies on sharing data and materials.” If there are restrictions on sharing of data and/or materials, please state these. Please note that we cannot proceed with consideration of your article until this information has been declared.**

**This information should be included in your cover letter; we will change the online submission form on your behalf.**

We have a provisional patent. There is no patent number as of yet. We will specify in the submission.

**5. In your Data Availability statement, you have not specified where the minimal data set underlying the results described in your manuscript can be found. PLOS defines a study's minimal data set as the underlying data used to reach the conclusions drawn in the manuscript and any additional data required to replicate the reported study findings in their entirety. All PLOS journals require that the minimal data set be made fully available. For more information about our data policy, please see** [**http://journals.plos.org/plosone/s/data-availability**](https://urldefense.com/v3/__http:/journals.plos.org/plosone/s/data-availability__;!!Cjnu1T6GFXg!5kUyB3tTfo_2QNeZFuMexc32vrxRJewf2OHX8H3xMvDnCc7i5AEkaKgEgkkLzbd8qv2uUQ$)**.**

**"Upon re-submitting your revised manuscript, please upload your study’s minimal underlying data set as either Supporting Information files or to a stable, public repository and include the relevant URLs, DOIs, or accession numbers within your revised cover letter. For a list of acceptable repositories, please see** [**http://journals.plos.org/plosone/s/data-availability#loc-recommended-repositories**](https://urldefense.com/v3/__http:/journals.plos.org/plosone/s/data-availability*loc-recommended-repositories__;Iw!!Cjnu1T6GFXg!5kUyB3tTfo_2QNeZFuMexc32vrxRJewf2OHX8H3xMvDnCc7i5AEkaKgEgkkLzbfCk5cOuA$)**. Any potentially identifying patient information must be fully anonymized.**

**Important: If there are ethical or legal restrictions to sharing your data publicly, please explain these restrictions in detail. Please see our guidelines for more information on what we consider unacceptable restrictions to publicly sharing data:** [**http://journals.plos.org/plosone/s/data-availability#loc-unacceptable-data-access-restrictions**](https://urldefense.com/v3/__http:/journals.plos.org/plosone/s/data-availability*loc-unacceptable-data-access-restrictions__;Iw!!Cjnu1T6GFXg!5kUyB3tTfo_2QNeZFuMexc32vrxRJewf2OHX8H3xMvDnCc7i5AEkaKgEgkkLzbfC_ZC8cw$)**. Note that it is not acceptable for the authors to be the sole named individuals responsible for ensuring data access.**

**We will update your Data Availability statement to reflect the information you provide in your cover letter.**

Our minimum data will be submitted with manuscript.

**6. Please include your full ethics statement in the ‘Methods’ section of your manuscript file. In your statement, please include the full name of the IRB or ethics committee who approved or waived your study, as well as whether or not you obtained informed written or verbal consent. If consent was waived for your study, please include this information in your statement as well.**

This has been updated.

**7. Please upload a copy of Figure 5, to which you refer in your text on page 17. If the figure is no longer to be included as part of the submission please remove all reference to it within the text**

We apologize greatly. Figure 5 was revised to Figure 4b and this reference was missed. It has been corrected.

**[Note: HTML markup is below. Please do not edit.]**

**Reviewer #1: Exline at al report on the application of a breath tester for the diagnosis of COVID19 in ICU patients. They found a specific breath print pattern (i.e. omega pattern) to predict COVID19 with 82% accuracy and they conclude that this technology could help for the rapid screening of COVID19.
On my opinion the main limitation of the study is that it is unclear to what extent the omega pattern really predicts SARS-CoV2 infection rather than lung or systemic inflammation, that is also known to have a significant impact on exhaled breath composition. In other words, the observed differences may be attributable to differences in the inflammatory responses of enrolled patients, and not to SARS-CoV2 presence. In addition to that, patients have been poorly characterized in term of other clinical and biochemical parameter (Table 1), and their association with the omega pattern has not been evaluated. Lastly, I do not see much interest in applying this test in ICU patients. I would be more interested in applying it in asymptomatic subjects for their early screening. In any case, results should be replicated in subjects without severe COVID19 infection, versus asymptomatic patients without COVID19.**

We agree in large part with reviewer #1’s second point concerning utility in the ambulatory settings for this technology. However, due to the novel nature of this technology and the logistical challenges of obtaining serial measurements in COVID positive patients in an outpatient setting, we chose to look at an ICU cohort initially. Further studies on this technology to demonstrate utility in other settings are ongoing.

To reviewer #1’s first point, we started with the ICU study to establish the form factor/breath print for COVID-19 by studying severely ill patients treated in the ICU with COVID-19 vs controls who were suffering from other infections, including pneumonia, UTI, etc. This manuscript focuses on the ICU study only. We have made a best effort to correlate the results of the breath test with clinical parameters that determine when lung or systemic inflammation has occurred. We are including more information about the pertinent false positive and false negative patients on day #1 to help readers see the utility of this technology.

**Reviewer #2: This is an interesting study in which a sensor was used to measure 'signatures' of NO and NH3 from the breath of COVID patients vs controls. I think this could eventually be published subject to some revisions.**

1. **The phrasing 'sensor array' is definitely not accurate. An array implies multiple different sensors operating in parallel, not a single catalytica sensor.**

We agree that the term sensor array is incorrect given that we utilize only one sensor for the output signal, and thus will be corrected in the revised manuscript.

2. **An area under the curve plot should be shown.**

Unfortunately, we are unable to apply a standard ROC curve to our study the output of the breathalyzer is qualitative rather than quantitative resulting in a dichotomous value that does not allow us to change the threshold value to develop a range of true negative and true positive values. To classify COVID, we performed classification of the waveform patterns by finding the extremum points not using a classifier. This results in 2 different looking curves. The omega pattern is defined as waveform with two minimum points and one maximum point (demonstrated in figure 3 of manuscript). The NO pattern is defined as waveform with one maximum point. The parameters used in the detection of the extremum points are the size of the neighborhood of the point that is examined and the normalized difference between the observed point and the ends of the neighborhood region. We then generate a sensitivity value by subtracting the maximum from minimum value, but given the differences in the curves we cannot compare the sensitivity from a patient with the omega pattern to a patient with the NO pattern.

3. **The raw sensor data for all patients should be shown. It is not clear what happens in cases of false positives and negatives.**

We will include our sensitivity data and characterizations in our minimum data. We will expand on the false positive / false negative cases in manuscript.

4. **The explanation of the omega pattern is possible but data supporting the explanation is minimal. Does introducing NO and NH3 to the sensor surface result in an omega pattern.**

This is an excellent question. We have produced a simulated device output that explains the mechanism accurately and will attempt to modify the manuscript to make this more clear that figure B below is a reproduction of omega. First, we demonstrated that ammonia is converted to NO on the sensor (figure from manuscript) shown below. Notice that at higher concentrations (> 2 ppm), ammonia is converted to NO by the sensor.

**Figure A.** NO, ammonia, isoprene, and acetone in a background of air were used as input in the breathalyzer device at 1000sccm flow rate

Next, we were able to reproduce the whole omega pattern by simulating the non-selective and selective oxidation of ammonia, i.e. N_2_ and NO, respectively (Figure B).

**Figure B.** Simulated conditions for the sensor response to breath exhaled by COVID patients.

**5. The speculation about having two sensors selective for ammonia and NO not working as well is not clear. I'd expect the more sensors the better the results.**

This is a good point. Additional sensors would allow for more information, however the more sensors mean that the relative ratio of the NO to ammonia needs to be recorded and that would be dependent on the stage of ammonia oxidation captured in a single measurement. Signal processing will not be able to determine a unique ratio of Ammonia to NO and extensive data analysis would be needed to determine a distinct profile of COVID based on such a ratio.

Having a single output (breath pattern/print) which is the temporal manifestation of the reactions between the key biomarkers (ammonia, NO, and O_2_) is invaluable as we know instantly that we have the signature for COVID. Thus one sensor allows rapid interpretation of the data without the need for additional signal processing or machine learning as is needed for electronic noses (see our response to 3 for more details on electronic noses).

We included a sentence in manuscript to help reader understand tradeoff between more sensors and rapidity of analysis.

**Reviewer #3: The work presents separation of patients with COVID-19 from patients with other diseases through measurements of nitric oxide (NO) and ammonia (NH3) in their breath.**

**1. Did you perform the tests at a certain time of the day, which might have affected the concentration of the volatiles that you detected? It would have been very informative if you could test daily variation of the volatiles (specifically the NO) and if it is different in patients compared to healthy individuals and/or other patient groups.**

This is an excellent observation. We are working on a prototype that would allow continuous monitoring to answer this question. Unfortunately, in the present study, we utilized once a day testing between approximately 8am and 2pm. This has been added to methods.

**2. How do you interpret those patients with COVID-19 that have rather constant omega amplitude at different days from the onset of disease, like the one indicated with red circles in Figure 4B?**

Great question. We think it represents continued inflammation as some of those patients remained on the ventilator for weeks post study. We attempted to add that to interpretation, but with caution as we do not have data that goes out that far.

**3. NO indication within "NO pattern" label at Figure 4B mixes up with nitric oxide, but it is not standing for that, as far as I understood. Also, does the "NO pattern" side of the figure in Figure 4B have negative sensitivity values? They are below zero, but the values are non-negative. Is it shown like that on purpose, or by mistake? If it is shown like that on purpose, it means that the sensitivity at the "omega pattern" side decreases with the progress of time and then it increases at the "NO pattern" side... Is it the case?**

That is the correct interpretation, the NO pattern sensitivity values are always positive. Waveform with NO pattern has a single maximum point and the sensitivity is defined as the normalized difference between the maximum point and the baseline. The sensitivities for two different waveform patterns, omega pattern and NO pattern, are shown on purpose on the same plot in order to demonstrate that as time progresses most of the patients go from being strongly ‘positive’ to ‘negative’. Starting from the day 1 of sampling the patient’s breath to day 10, the sensor response for most of the patients adheres to the following trend: the response is initially a ‘strong positive’ with omega pattern and high sensitivity value, then still with the omega pattern sensitivity reduces, that is the patient becomes less ‘positive’. As time passes, the pattern shifts to NO pattern with increasing sensitivity value, that is the patient becomes more ‘negative’ with time. We have attempted to alter the manuscript to make that more clear.

**4.It is written on page 5, line 66 that “The technology presented here is distinct from other artificial olfactory systems..." Please cite the respective studies there.**

Thank you for this question, we have an extensive explanation below as this is clearly near and dear to our hearts, but in the interest of brevity for the manuscript we linked to the ACS Nano study and a briefer description for readers on p5.

Electronic noses attempt to mimic the human olfactory system by utilizing an array of non-selective chemical (gas) detectors (from a few to the hundreds). The e-nose technology originates from cheese, wine and perfume Odor detection (Noses); They can be desktop size or portable; They all require complex signal processing; The cost of these devices varies according to the type of sensor technology used and the complexity of the analysis involved; The lack of relative specificity to a mixture of gaseous analytes for these sensing elements makes necessary the use of machine learning algorithms.

Our sensor technology is distinct from the electronic nose technology which is typically described in *ACS Nano 2020, 14, 12125−12132* . According to this publication: *An array consisting of eight gold nanoparticles linked to organic ligands, creating a diverse sensing layer that can swell or shrink upon exposure to volatile organic compounds (VOCs), causing changes in the electric resistance. When exposed, VOCs diffuse into the sensing layer or fall on the sensing surface and react with the organic segment or the functional groups capping the inorganic nanomaterials. The outcome of the interactions causes a swift charge transfer to/from the inorganic nanomaterial, producing variations in the measured conductivity. Different sensors can be used, as an array of cross-reactive semiselective sensory units that mimic the sensing processing method of natural mammalian olfactory system. The sensor array was integrated with electronic circuitry and an advanced apparatus that collects an exhaled breath sample. A built-in sensor technology advised the study subject when the test was complete. Software-based machine learning methods probe the pattern of output signals to get a signature of COVID-19.*

So, the e-nose technology utilizes non-selective sensors in an array-these need to be calibrated every time they are used in a different environment. They swell and shrink to change the electrical properties of a transducer. They look for VOCs in general and need advanced machine learning and feature extraction algorithms to separate the signal.

Our technology utilizes a single metal oxide sensor that is selective to NO and it is catalytically active and within 15 seconds gives a distinct pattern of the disease based on the detection of the key biomarker of COVID-19-that is ammonia. We know what we look for and measure it. Ours is an advanced yet inexpensive sensor technology. It originates from automotive gas sensors for monitoring exhaust gases and CO detectors. It utilizes gas-selective materials to detect specific biomarkers signaling disease. It is available as portable, hand-held, or even wearable devices. This is a disruptive sensing technology which will allow individuals to monitor their health, detect diseases early, and control the effectiveness of their therapeutic treatment. It can be a diagnostic as well as a screening tool.

**5.Inclusion to the study required mechanical ventilation (page 6, line 104) and exclusion required mechanical ventilation more than 72 hours (page 6, line 106). So, did inclusion require mechanical ventilation up to 72 hours? What were the exact criteria? Did you include those that require mechanical ventilation only up to 3 days and collected sample on days 1, 3 (days during mechanical ventilation), and 7 and 10 (days after mechanical ventilation)? That issue seems to require clarification...**

Patients had to be on mechanical ventilation for eligibility. Only patients that had been on a ventilator for less than 72-hours at time of enrollment were eligible, essentially you were eligible from 0-72 hours after initiation of ventilation. Post-enrollment we followed with sample collections for up to 10 days. We have clarified this in the methods section.

**6.About the reaction equation written on page 8, lines 138-139: As far as I know, you cannot write non-integer numbers as the number of molecules entering a reaction or the number of molecules generated as products. In relation, does NH3 oxidize to NO, or reacts with NO as shown in lines 138-139? Both are mentioned in the text. Do both occur in this sensor? Besides, the reaction equation written on page 8, lines 138-139, is not present in the cited reference (reference number 10, Gouma et al. 2010 IEEE Sensors J 10:49) as far as I have seen.**

Thank you for this correction. We have corrected the formula and reference. The oxidation of ammonia to NO occurs on the sensor. However, before reaching the sensing chamber, NH3, NO amd O2 are reacting following the equation below, which has been corrected to include full molecules:

**4NH3 + 6NO =**  **5N2+6H2O**

As mentioned in the manuscript, “**This reaction is responsible for the first reducing step (possibly catalyzed by the Pt electrodes of the heater)** observed as soon as the exhaled breath reaches the sensor, which lowers the baseline resistance of the sensor which reaches a minimum (S1). This step has been simulated in figure B above-(air off/N_2_ on). Next there is an oxidative step (S2 peak) corresponding to ***ammonia oxidation to NO which happens on the sensor***

As far as the reference, the original reference with formula is: *Platinum Metals Rev.*, 1960, **4**, (4), 122 Platinum Catalysts in Ammonia Oxidation, By B. H. J. Bell

**7. It is written on page 8, lines 146-147 that "...these redox processes on the sensor are not favored at temperatures below 300°C." What was the measurement temperature?**

The operating temperature was 300°C. We reference that now in manuscript to ensure readers understand implications of the change in temperature.

**8. It is written on page 8, lines 149-150 that WO3 "... act both as sensor and as a catalyst for redox reactions involving ammonia and NO in air." Do you mean NO in the air or in the exhaled breath there?**

We mean NO in exhaled breath. We will specify it in the revised manuscript.

**9. Figure 5 is cited at two places in the text. However, there is no Figure 5 or its caption, please add that. Also, can you comment on the observation mentioned on page 9, lines 172-173, i.e., high NO in breath samples of patients with other diseases than COVID-19?**

We apologize for this oversite in our revising of manuscript. It should read Figure 4B instead of 5. We will correct it in the revised version. The line mentioned above was misleading, it should have specified patients with NO (non-COVID) pattern had high sens (see discussion on point #3 above). This line has been deleted to improve clarity.

**10. It is written on page 9, lines 176-177 that "...seven patients’ data was excluded due to us failing to detect the end-of-the-life of the sensor on time..." Did you lose the collected data, or else?**

The sensor has a limited lifetime as the baseline of the sensor drifts due to the contamination of the sensor surface. As the sensor’s baseline becomes high, the sensor stops responding to NO. Monitoring the baseline of the sensor prior to the measurement detects the failure of the sensor and the sensor is replaced with the new one. As we started experiments, we didn’t incorporate the end-of-lifetime detection. Samples were analyzed for seven patients with the sensor that reached the end of the life. Due to the time frame between the sensor dying and reviewing the data and the blinding of the lab personal we were unaware this occurred until our interim data review. This is the reason why data collected from these patients was excluded and the end-of-the-life detection has been incorporated from that moment in the software. We have explained this more fully in the discussion / limitations.

**11. Was the higher incidence rate of diabetes in the COVID group related to its being a risk factor?**

Great point. Yes, likely both obesity and diabetes were higher in the COVID population due to risks associated with poor COVID-19 outcomes. We have included that in results now.

**12. It is written on page 11 lines 204-206 that "Most well known include single selective sensors for each specific biomarker, e.g. NO for asthma, or acetone for metabolic disorders." Please cite the relevant references there.**

This was confusing on our part, the references included in the previous sentence had these topics covered, but we expanded and specified which references were for NO for asthma and actone in revised manuscript.

**13.Please check reference 9, which is cited for the DRIFTS study on page 8, line 154, and for a clinical study, earlier on page 7, line 113. It is not involving DRIFTS study. Details of reference 9 that is given at the references section: Lambden S, Laterre PF, Levy MM, Francois B. The SOFA score-development, utility and challenges of accurate assessment in clinical trials. Crit Care. 2019;23(1). doi: ARTN 374 10.1186/s13054‐019‐2663‐7. PubMed PMID: WOS:000501779700001.**

This was an error in our footnoting and has been corrected to appropriate study.

I. Jimenez, M.A. Centeno, R. Scotti, F. Morazzoni, A. Cornet, and J.R. Morante, “NH_3_ interaction with catalytically modified Nano-WO_3_ Powders for Gas sensing Applications”, J. Electrochem. Soc., 150(4), H72-H80, 2003.

**14. Please include a discussion including comparison of your sensor with the other breath tests that are developed for COVID-19, and for testing the same analytes.**

This is also a great interest of ours. Please see response to comment #4 above. The competitive technologies look for the overall VOC signature, but this can be misleading as many of the COVID infected patients will have high acetone levels due to metabolic disorders related to diabetes. Our sensor does not detect acetone or any by products of its oxidation.

Furthermore, GC-MS techniques that are usually employed to detect biomarkers in breath cannot detect ammonia (see ref: *ACS Energy Lett.* 2020, 5, 12, 3773–3777)

The problems with ammonia detection using GC-MS: “Ammonia gas is well-known for its tendency to physisorb onto most materials, including stainless steel tubing, often used to connect experimental setups to the GC inlet and sometimes also for GC internal connections. Physisorption onto tubing and valves becomes a very relevant issue when ammonia concentration approaches the sub-ppm range, as most analyte would be lost because of physisorption, with drastic implications for quantitative detection.”

Therefore, our approach is unique and highly reliable. We tried to include more of this discussion in manuscript.

15.

**About language and other issues:
Please check the language.
Breath bad is written at the caption of Figure 1. Is it correct, or did you mean breath bag?
Please indicate at axis labels of Figure 3 that time is in seconds.
Please correct Ieee as IEEE on page 15, line 293.
Also, please write the full-length form of abbreviations where they first appear in the text and at the caption of figures and tables, when abbreviations are present in tables and figures.
COVID needs to be replaced with COVID-19.**

We apologize for these mistakes. We have corrected the grammatical and spelling errors to the best of our ability.
